# Exercise-Generated β-Aminoisobutyric Acid (BAIBA) Reduces Cardiomyocyte Metabolic Stress and Apoptosis Caused by Mitochondrial Dysfunction Through the miR-208b/AMPK Pathway

**DOI:** 10.3389/fcvm.2022.803510

**Published:** 2022-02-25

**Authors:** Yanan Yu, Wewei Chen, Ming Yu, Jinsha Liu, Huan Sun, Ping Yang

**Affiliations:** ^1^Department of Rehabilitation, China-Japan Union Hospital, Changchun, China; ^2^Jilin Provincial Engineering Laboratory for Endothelial Function and Genetic Diagnosis of Cardiovascular Disease, Jilin Provincial Cardiovascular Research Center, Changchun, China; ^3^Department of Cardiology, China-Japan Union Hospital, Changchun, China

**Keywords:** mitochondrial dysfunction, metabolic stress, exercise, lipid metabolism, heart failure

## Abstract

**Objective:**

To explore the cardioprotective effects of exercise-derived β-aminoisobutyric (BAIBA) on cardiomyocyte apoptosis and energy metabolism in a rat model of heart failure (HF).

**Methods:**

In male Sprague-Dawley rats (8-week-old), myocardial infarction (MI) was used to induce HF by ligating the left anterior descending branch of the coronary artery. In the Sham group, the coronary artery was threaded but not ligated. After HF development, Sham and HF rats were exercised 60 min daily, 5 days/week on a treadmill for 8 weeks (50–60% maximal intensity) and exercise-induced cardiac remodeling after MI were assessed using echocardiography, hematoxylin and eosin (H&E), Masson's Trichrome, and TUNEL staining for the detection of apoptosis-associated factors in cardiac tissue. High-throughput sequencing and mass spectrometry were used to measure BAIBA production and to explore its cardioprotective effects and molecular actions. To further characterize the cardioprotective effects of BAIBA, an *in vitro* model of apoptosis was generated by applying H_2_*O*_2_ to H9C2 cells to induce mitochondrial dysfunction. In addition, cells were transfected with either a miR-208b analog or a miR-208b inhibitor. Apoptosis-related proteins were detected by Western Blotting (WB). ATP production was also assessed by luminometry. After administration of BAIBA and Compound C, the expression of proteins related to apoptosis, mitochondrial function, lipid uptake, and β-oxidative were determined. Changes in the levels of reactive oxygen species (ROS) were assessed by fluorescence microscopy. In addition, alterations in membrane potential (δψm) were obtained by confocal microscopy.

**Results:**

Rats with HF after MI are accompanied by mitochondrial dysfunction, metabolic stress and apoptosis. Reduced expression of apoptosis-related proteins was observed, together with increased ATP production and reduced mitochondrial dysfunction in the exercised compared with the Sham (non-exercised) HF group. Importantly, exercise increased the production of BAIBA, irrespective of the presence of HF. To assess whether BAIBA had similar effects to exercise in ameliorating HF-induced adverse cardiac remodeling, rats were treated with 75 mg/kg/ day of BAIBA and we found BAIBA had a similar cardioprotective effect. Transcriptomic analyses found that the expression of miR-208b was increased after BAIBA administration, and subsequent transfection with an miR-208b analog ameliorated both the expression of apoptosis-related proteins and energy metabolism in H_2_O_2_-treated H9C2 cells. In combining transcriptomic with metabolomic analyses, we identified AMPK as a downstream target for BAIBA in attenuating metabolic stress in HF. Further cell experiments confirmed that BAIBA increased AMPK phosphorylation and had a cardioprotective effect on downstream fatty acid uptake, oxidative efficiency, and mitochondrial function, which was prevented by the AMPK inhibitor Compound C.

**Conclusion:**

Exercise-generated BAIBA can reduce cardiomyocyte metabolic stress and apoptosis induced by mitochondrial dysfunction through the miR-208b/AMPK pathway.

## Introduction

Metabolic disorders play a key role in the occurrence and progression of heart failure (HF) after myocardial infarction (MI). The restoration of energy homeostasis is important for cardiac function (1). Mitochondria produce ATP and can thus regulate energy metabolism (2), which involves the selection and utilization of substrates, efficient oxidative phosphorylation, and energy shuttling at the mitochondrial level (3). These metabolic processes are closely integrated to maintain the rapid, sustained, and sufficient output of ATP. HF leads to a loss of metabolic substrate flexibility and a decrease in oxidative phosphorylation efficiency, which is accompanied by mitochondrial dysfunction (4, 5). This metabolic dysregulation can occur due to destruction of the mitochondrial complex, mitochondrial uncoupling, crest remodeling and swelling, and changes of mitochondrial membrane potential, leading to reactive oxygen species (ROS) accumulation, insufficient ATP production, apoptosis, and death (6–8). Thus, finding ways to attenuate cardiac metabolic stress and mitochondrial dysfunction in patients with HF is of clinical importance.

Of the known interventions for HF patients, moderate exercise is known to have a beneficial effect on cardiac function in this population (9–14). Indeed, moderate continuous endurance training is known to be a safe, efficient, and well-tolerated intervention by HF patients that it is recommended by the Heart Failure Association (15). Importantly, impairments of cardiac metabolism in HF patients can be partially restored through exercise due to the improved mitochondrial quality control, bioenergetics, and cardiac function (16). Despite the known beneficial effects of exercise in HF patients, many HF patients are exercise intolerant or have a reduced exercise capability (17, 18). Therefore, being able to identify a substance that can mimic the beneficial effects of exercise in improving cardiac function and energy metabolism in patients with HF is necessary. BAIBA is a small molecule (103.6 Da) composed of two enantiomers (L-BAIBA and D-BAIBA) produced by skeletal muscles during exercise, and mediates the beneficial effect of exercise from skeletal muscle to other tissues and organs via the endocrine system (19). In this study, we have focused mainly on L-BAIBA, which is produced by muscle contraction (19) and can effectively improve glucose and lipid metabolism, activate β-oxidation of fatty acids in the liver, and increase AMPK phosphorylation in adipose tissue (20–25). However, whether BAIBA could be used a cardioprotective therapeutic intervention to mimic the effects of exercise in patients with HF has not been studied.

Our study found that exercise-generated BAIBA could effectively improve cardiac function, energy metabolism, and reduce apoptosis in rats with HF after MI. Importantly, oral administration of BAIBA increased the expression of miR-208b, which is a heart-specific miRNA that is encoded on intron 27 of the gene encoding α-MHC and is essential for the expression of genes involved in cardiac contractility and fibrosis. These findings align with previous studies from our group (26), suggesting miR-208b has a protective effect on myocardial apoptosis and mitochondrial dysfunction in rats with MI. Moreover, BAIBA also increased the ratio of p-AMPK/AMPK in HF rats, reducing apoptosis in H_2_O_2_-induced H9C2 cells and leading to improved mitochondrial function and energy metabolism, suggesting BAIBA reduces cardiomyocyte metabolic stress and apoptosis caused by mitochondrial dysfunction through the miR-208b/AMPK pathway.

## Materials and Methods

### Animal Experiments

Animal experiments were approved by the Ethics Committee of the First Hospital of Jilin University. Male Sprague-Dawley (SD) rats (8 weeks, 250 g) were used to establish. In male Sprague-Dawley rats (8-week-old), myocardial infarction (MI) was used to induce HF by ligating the left anterior descending branch of the coronary artery (27). In the Sham group, the coronary artery was threaded but not ligated. The procedure was carried out under aseptic conditions and anesthesia induced by inhaling oxygen anesthesia containing 1.5–2.0% isoflurane. After disinfection, the skin of the prothoracic area was cut longitudinally, the chest muscle was separated, the intercostal space was exposed and punctured, and the anterior descending branch of the coronary artery was ligated after separating the pericardium. The soft tissue and skin of the chest was sutured and disinfected with penicillin. Intraperitoneal injections of 160,000 units/day penicillin were administered for a total of seven days. Four weeks after the procedure, the rats were examined by echocardiography and divided into separated groups according to ejection fraction (EF) %. Details of the groups are shown in Supplementary File 1. Briefly, for the exercise studies, rats were divided into 4 groups: (i) Sham; (ii) heart failure (HF); (iii) heart failure with exercise (HFE); and (iv) Sham and exercise (ShamE) groups. For the β-aminoisobutyric (BAIBA) studies, rats were divided into 3 groups: (i) Sham; (ii) heart failure (HF); (iii) heart failure with BAIBA(BAIBA) groups. For the exercise groups, rats were exercised daily on a treadmill (Shanghai Ruanlong Technology Co., Ltd. BW-ZHPT) involving 10–20 min acclimation, 0.3–0.6 km/h, and 0% incline. After 3 days' acclimation, the maximum exercise intensity was tested, starting at 0.3 km/h and increasing by 0.3 km/h every 3 min until exhaustion (28). The rats were run on the treadmill for 8 weeks (50 to 60% of maximum intensity), 60 min/day, 5 days/week, and at a 0% incline. For the BAIBA studies, BAIBA (MCE; HY-113380) was administered orally by gavage at a daily dose of 75 mg/kg for 8 weeks (29, 30) to rats with HF. The corresponding dose of BAIBA was dissolved in 1ml ddH2O, and the rats in the Sham group and the HF group were given the same volume of ddH2O orally by gavage. At 12 weeks post-op, rats in each group were examined by echocardiography, and the hearts were quickly removed under deep anesthesia. After the heart was removed, it was washed with cold PBS, dried with sterile medical gauze. Part of the left ventricle was quickly fixed in 4% paraformaldehyde for subsequent histological evaluation, while part of the left ventricle was quickly frozen in liquid nitrogen (or at−80°C) for subsequent transcriptomic, proteomic, and biochemical analysis. All experiments were conducted following the guidelines of animal research institutions and in accordance with the guidelines for the Care and Use of Experimental Animals published by the National Institutes of Health (NIH publ.no.85-23, revised in 1996).

### Echocardiography

To non-invasively assess changes in cardiac structure and function, GE VIVId-i was used for the echocardiographic examinations at 4 and 12 weeks post-MI. Rats were anesthetized using the method used for the infarction surgery (described above) and were maintained under anesthesia for the entire echocardiographic examination. The probe (10S; 4.0–11.0 MHz) was placed directly on the shaved chest wall. The left ventricular end diastolic diameter (LVIDd), left ventricular end systolic diameter (LVIDs), interventricular septal thickness (IVDs), ejection fraction (EF) % and fractional shortening (FS) were measured. EF was calculated as stroke volume (SV)/end-diastolic volume (EDV) × 100%. FS was calculated as (LVIDd–LVIDs)/LVIDd × 100%.

### H&E Staining

The left ventricular tissue was fixed in 4% paraformaldehyde for 24 h, embedded in paraffin, and sliced into 4 μm sections. The sections were dewaxed with xylene and an ethanol gradient, stained with hematoxylin, washed with 1% (v/v) hydrochloric acid for 30 s, and stained with eosin for 2 min. After gradient ethanol dehydration, permeation, and sealing, photographs were taken under a microscope (OLYMPUS BX53).

### Masson's Staining

Paraffin sections were dewaxed into the water, stained with Wiegert's iron hematoxylin solution for 5 min, differentiated with hydrochloric acid alcohol, and washed with tap water. The sections were stained with fuchsin solution for 5 min, molybdophosphoriTUNEL acid solution for 3 min, and toluidine blue-O re-staining for 5 min. The slices were soaked in 1% glacial acetic acid for 1 min before dehydration, permeation, and sealing. Sections were observed and photographed under a microscope (OLYMPUS BX53). Image-Pro Plus 6.0 software (Media Cybernetics, USA) was used to analyze images. Three visual fields at x400 were tested randomly in each sample. The degree of fibrosis was calculated as the percentage of collagen area.

### TUNEL Staining

Paraffin sections were dewaxed with xylene, hydrated with an ethanol gradient, and then treated with 20 μg /ml protease KJI without DNase at 37°C for 30 min. After repeated rinsing with PBS, 50 μl TUNEL monitoring solution was added and the sample was incubated at 37°C for 60 min. After washing, the sections were sealed with an anti-fluorescence quenching solution and observed under a fluorescence microscope (Lycra, DL-80, Germany). Nuclei of normal and apoptotic cells appeared light blue and light red after staining, respectively. Three visual fields at x400 were tested randomly in each sample. The apoptotic index value was calculated as the number of apoptotic cells divided by the total number of cells in each field of view (x400).

### Transmission Electron Microscopy

Mitochondrial ultrastructure was observed by TEM. Tissue specimens were fixed with 3% glutaraldehyde, then re-fixed in 1% SO4, rinsed with PBS solution, and embedded in Epon resin after ethanol gradient dehydration. Ultra-thin slices (50 nm) were cut using a diamond knife, and the glass slides were fixed onto the copper specimen table with conductive paste. Platinum was sprayed onto the sample for transmission electron microscopic observation (Hitachi H600 Electron Microscope, Hitachi, Japan) and imaging. Magnification is × 1.2k; × 3.0k, and × 8.0k, respectively.

### Cell Transfection

Rat cardio myoblast H9C2 cells were cultured until 60–70% confluent and were then transfected using Lipofectamine 2000 (Invitrogen, Carlsbad, CA, USA) with a miR-208b analog (100 nM), a miR-208b inhibitor (100 nM), or a miR-208b negative control (NC) vector (100 nM) in serum-free and Penicillin-Streptomycin-free medium, after 6 h, the cells were changed into complete medium and continued to be cultured for 24 h before incubation with H_2_O_2_. The miR-208b analog, miR-208b inhibitor, and miR-208b NC were designed by Gene Pharma (Suzhou, China). The sequences were as follows: miR-208b analog; (5′-AUAAGACGAACAAAAGGU-3′ 5′-CUUUUGUUCGUCUUAUUU−3′), miR-208b inhibitor; (5′-ACCUUUUGUUCGUCUUAU-3′), miR208b Negative control (NC); (5′-CAGUACUUUUGUGUAGUACAA-3′).

### Cell Experiments

Mitochondrial dysfunction was induced in H9C2 cells by incubation with 200 μM H_2_O_2_ for 24 h to generate a model of myocardial apoptosis. The cells were divided into the following groups: Control, H_2_O_2_, BAIBA, miR-208b analog, miR-208b inhibitor, miR-208b NC, and Control, H_2_O_2_, BAIBA, Compound C. The transfection concentration of miR-208b was 100 nM. The concentrations of BAIBA and Compound C were 30 and 10 μM, respectively.

### Western Blotting

H9C2 cells or myocardial tissue were treated with RIPA lysis buffer containing protease and phosphatase inhibitors (Sangon Biotech, Shanghai, China), and total protein concentrations were measured using a BCA protein assay kit (Sangon Biotech, Shanghai, China). After denaturation at 95°C for 5 min in SDS sample buffer, proteins were separated on SDS-PAGE and transferred to PVDF membranes. The membranes were blocked with 5% (w/v) BSA for 1 h, followed by incubation overnight at 4°C with specific antibodies (all from Abcam, Cambridge, UK) against the following proteins: Bax (ab32503), BCL-2 (ab196495), pro-caspase 3(ab184787), cleaved-caspase 3 (ab214430), DRP-1 (ab184247), OPA-1 (ab42364), PGC-1 α (ab106814), SUCLA (ab202582), ACADL (ab129711), CD36 (ab133625), CPT-1 (ab234111), P-ACC (ab68191), ACC (ab45174), p-AMPK (ab133448), AMPK (ab207442), and GAPDH (ab181602). After washing, the membrane was incubated with an HRP-coupled secondary antibody for 60 min, washed again, and finally incubated with enhanced chemiluminescence (ECL) solution (Sigma, USA).

### qRT-PCR Analysis

Myocardial tissue samples or H9C2 cell samples were homogenized in TRIzol reagent for RNA extraction and the RNA concentration was evaluated by spectrophotometry. Equal amounts (2 μg) of purified RNA were used as templates, and the First Strand cDNA synthesis kit (Sangon Biotech, Shanghai, China) was used to synthesize cDNA and miRNA. Quantitative RT-PCR analysis was performed on a real-time PCR system (ABI 7500 DX) using SYBR Green Master Mix (Sangon Biotech), cDNA template, and specific primers (miR-208-3p primer 5′-GCGCATAAGACGAACAAAAGGT-3′). GAPDH was used to standardize mRNA expression, while U6 (also known as RNU6) was used to standardize miRNA expression. The relative expression levels of the target genes were determined by the 2^−ΔΔCT^ method.

### ATP Measurements

An ATP test kit (Beyotime, Shanghai, China) was used according to the manufacturer's instructions. Cell lysates or myocardial tissue lysates were centrifuged at 12 000 x g for 5 min, and the supernatant was retained. One hundred microliters of the supplied ATP solution were added to each of the wells of 96-well plates and allowed to stand at room temperature for 3–5 min. Twenty microliters of the lysate supernatant or standard solutions were then added, mixed quickly, and, after 5 sec, the LUL value (relative light unit, RLU) was determined by chemiluminescence measurement in a luminometer.

### Mitochondrial Membrane Potential

JC-1 dye (5,5', 6,6'-tetrachloro-1,1', 3,3'-tetraethyl Benzimidazole carbonyl cyanine iodide) was used to evaluate Δ ψ m. H9C2 cells were incubated with JC-1 staining solution at 37°C for 20 minutes. The cells were then washed with JC-1 buffer and imaged by confocal microscopy (Nikon, A1, Japan). JC-1 can form aggregates in healthy mitochondria and has red fluorescence (Em. 590 nm) at the polarized Δ ψ m. In cells with altered mitochondrial function, JC-1 can only form a monomer with resultant green fluorescence (Em. 527 nm) in the cytoplasm during depolarization. The ratio between red and green fluorescence of H9C2 cells was used for the detection of the mitochondrial membrane potential.

### Detection of Intracellular ROS Level

The production of ROS was measured using a ROS assay kit (Beyotime) using the 2-dichlorofluorescein diacetate (H_2_-DCFDA) method. H9C2 cells were incubated with 10 μM H_2_-DCFDA in the dark at 37°C for 20 min. After washing with PBS, the cells were imaged with fluorescence microscopy (Leica DL-80, Germany).

### Non-targeted Metabolomics Sequencing

Rat myocardial tissues were used for non-targeted metabolomics sequencing. The specific location was the anterior wall of the left ventricle (in the BAIBA group, the area around the infarction was used). One milliliter of 90% cold methanol was added to 100 ml of sample, which was then homogenized using an MP homogenizer followed by sonication at low temperature and centrifugation. The supernatant was dried in a vacuum centrifuge. For LC-MS analysis, the samples were re-dissolved in 100 μL acetonitrile/water (1:1, v/v). For HILIC separation, samples were analyzed using a 2.1 x 100 mm ACQUIY UPLC BEH 1.7 μm column (Waters, Ireland). A 2 μL aliquot of each sample was injected. In the MS-only acquisition, the instrument was set to acquire over the m/z range 60–1000 Da. The product ion scan was acquired using information-dependent acquisition (IDA) with selection of the high-sensitivity mode. The raw MS data (wiff.scan files) were converted to MzXML files using ProteoWizard MSConvert before importing into the freely available XCMS software. In the extracted ion features, only the variables with over 50% of the non-zero measurement values in at least one group were kept. Compound identification of metabolites in the MS/MS spectra was performed with an in-house database established with available authentic standards. After normalization to total peak intensity, the processed data were uploaded before importing into SIMCA-P (version 16.1, Umetrics, Umea, Sweden), where the data were subjected to multivariate data analysis, including Pareto-scaled principal component analysis (PCA) and orthogonal partial least-squares discriminant analysis (OPLS-DA). Seven-fold cross-validation and response permutation testing was used to evaluate the robustness of the model. The variable importance in the projection (VIP) value of each variable in the OPLS-DA model was calculated to measure its contribution to the classification. Metabolites with VIP values >1 was further analyzed for significance using Student's *t*-test at the univariate level, with *p*-values < 0.05 considered as statistically significant.

### Transcriptomics Sequencing

Rat myocardial tissues were used for non-targeted metabolomics sequencing. Specifically, the anterior wall of the left ventricle was used; in the BAIBA group the area around the infarction was used). Total RNA was extracted using the TRIzol® reagent. Paired-end libraries were prepared using an ABclonal mRNA-seq Lib Prep Kit (ABclonal, China). Adaptor-ligated cDNAs were used for PCR amplification. The PCR products were purified (AMPure XP system) and library quality was assessed on an Agilent Bioanalyzer 4150 system. Finally, sequencing was performed with an Illumina Novaseq 6000 /MGISEQ-T7 system. The data generated from Illumina/BGI platform were used for bioinformatics analysis. All analyses were performed using an in-house pipeline from Shanghai Applied Protein Technology. In this step, the adapter sequences were removed and low-quality sequences and reads with N ratios > 5% were filtered out to obtain clean reads for subsequent analysis. The clean reads were aligned separately to the reference genome with orientation mode using HISAT2 software to obtain mapped reads. The mapped reads were spliced using Stringtie software, after which Gffcompare software was used to compare them with the reference genome GTF/GFF file to identify the original unannotated transcription region and discover new transcripts and new genes. Feature Counts was used to count the reads numbers mapped to each gene. The FPKM of each gene was calculated based on the length of the gene and reads count mapped to the gene. Differential expression analysis was performed using the DESeq2, and genes with |log2FC|>1 and Padj < 0.05 were considered to be differentially expressed genes.

### Joint Analysis of Sequencing Data

Principle component analysis (PCA) was performed with SIMCA Version 14.1 using quantitative data from the two omics analyses. All differentially expressed genes and metabolites were queried and mapped to pathways based on the online Kyoto Encyclopedia of Genes and Genomes (KEGG, http://www.kegg.jp/). Enrichment analysis was also performed. R Version 3.5.1 was used to combine the KEGG annotation and enrichment result of the omics analyses. Venn diagrams and bar plots were drawn. Z-score normalization was performed on the quantitative data of the target differentially modified peptides, and heatmaps were drawn with R Version 3.5.1 (Distance Matrix Computation: Euclidean, Hierarchical Clustering: complete linkage).

### Statistical Analysis

GraphPad Prism5 software (GraphPad Software Company, San Diego, CA, USA) was used to determine independent-sample differences between two groups. The differences between multiple groups were tested by one-way ANOVA. *P*-values < 0.05 were considered statistically significant.

## Results

### Exercise Improves Cardiac Morphology and Function in Rats With Heart Failure After Myocardial Infarction

**Figure 1:**
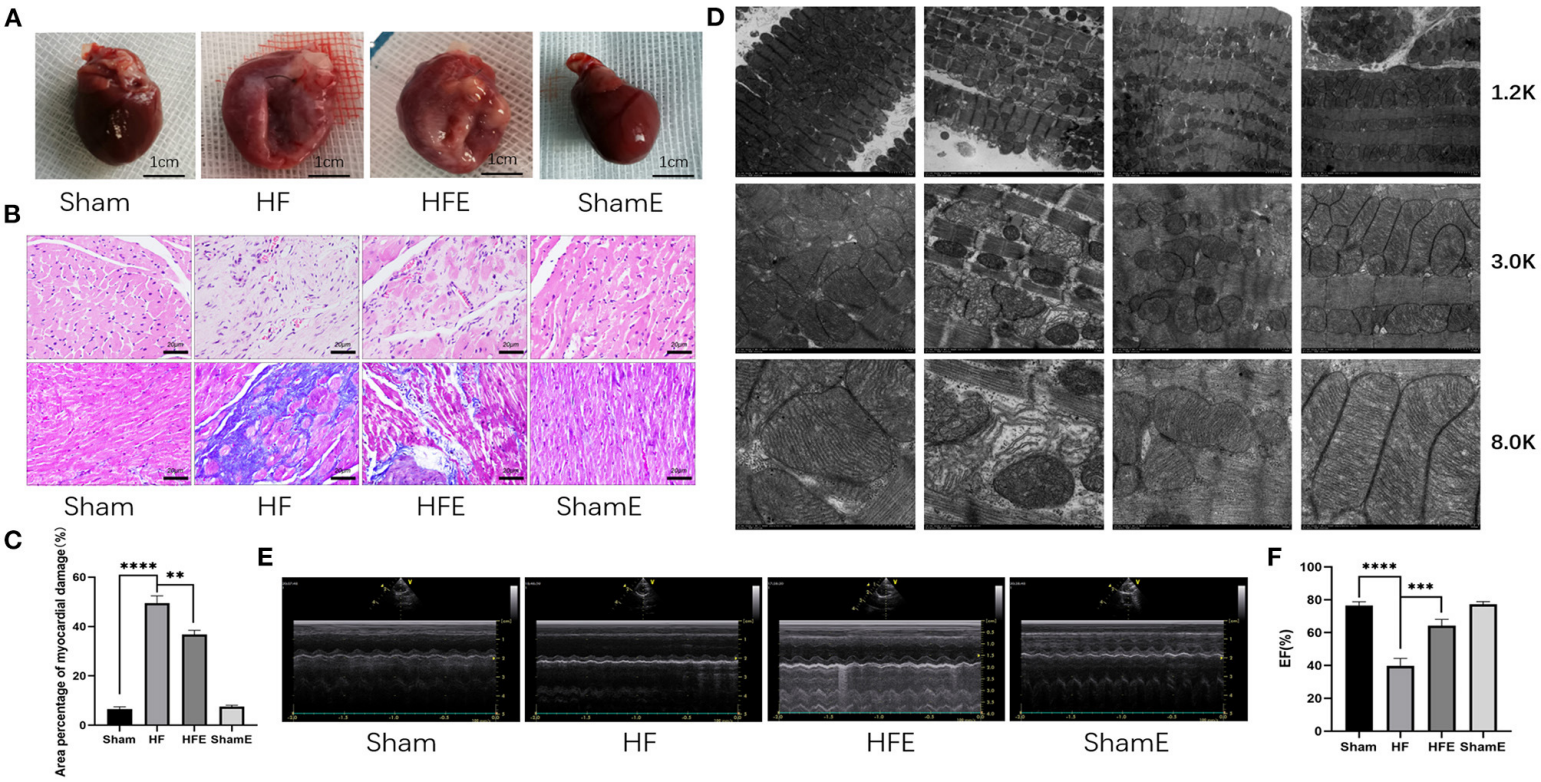
Exercise improves cardiac morphological changes and function in rats after induced myocardial infarction. **(A)** The overall appearance of the hearts of rats from the Sham, HF, HFE, and ShamE groups, scale bar = 1 cm. **(B)** Cardiomyocytes stained by H&E, Scale bar = 20 μm, Mason tricolor-stained cardiac fibrosis, scale bar = 20 μm. **(C)** Quantitative analysis of collagen deposition in the left ventricle; *n* = 3; ****P < 0.0001; **P < 0.01. **(D)** Electron micrograph (TEM) of mitochondrial morphology in a longitudinal section of ventricular muscle. Magnification, × 12 000; × 30 000, and × 80 000. **(E)** M-mode echocardiography of rats in each group. **(F)** Statistical analysis of left ventricular EF determined by echocardiography. *n* = 6; ****P < 0.0001; ***P < 0.001.

We established the rat model of HF after myocardial infarction (MI) by ligation of the anterior descending coronary artery. The immediate mortality rate of MI group was 61%, and the delayed mortality rate was 63.9%. It is well-established that MI induces maladaptive changes in the myocardium and extracellular matrix (ECM), which leads to ventricular pathological remodeling (structural and functional) and the occurrence and development of heart failure (HF) (Figure 1). Indeed, compared with rats in the Sham group, rats in the HF group showed morphological abnormalities, such as incomplete structure, different size, and disordered arrangement of cardiomyocytes (Figure 1A). By contrast, we found that 8 weeks of treadmill exercise (50 to 60% of maximum intensity, 60 min/day, 5 days/week, and at a 0% incline) reduces adverse MI-induced morphological changes. Specifically, despite the presence of a clear ischemic necrotic area in MI compared with the Sham group, the anterior wall of the left ventricle was still able to support and maintain the normal shape of the left ventricle (Figure 1A). Moreover, as shown in (Figures 1B,C), large collagen deposits were seen in the intercellular spaces, together with damaged cells and red blood cells, among other abnormalities with HF, which was ameliorated with exercise (*P* = 0.008). We further observed the morphology of mitochondria in the peri-infarcted area of rats in each group. Compared with the Sham group, the sarcoplasmic reticulum in the HF group appeared dilated, and the transverse canal (TT) was compressed and deformed (Figure 1D). Moreover, the mitochondria were arranged in a disordered fashion, with swollen mitochondrial crests and many absent cristae, and there was significant mitochondrial vacuolation. Surprisingly, exercise reduced mitochondrial vacuolation in the peri-infarcted area of the HFE group, and crest structures appeared relatively regular, suggesting exercise improved cardiac morphological changes and function after MI (Figure 1D). Consistent with this contention, echocardiography revealed that compared with the Sham group, LVIDd and LVIDs in HF group had increased, while EF, FS, and IVDs was significantly decreased, which was ameliorated with exercise in the HFE group (Figures 1E,F, Supplementary Files 3A–D).

### Exercise Alleviates Apoptosis in Rats With Heart Failure After Myocardial Infarction

**Figure 2:**
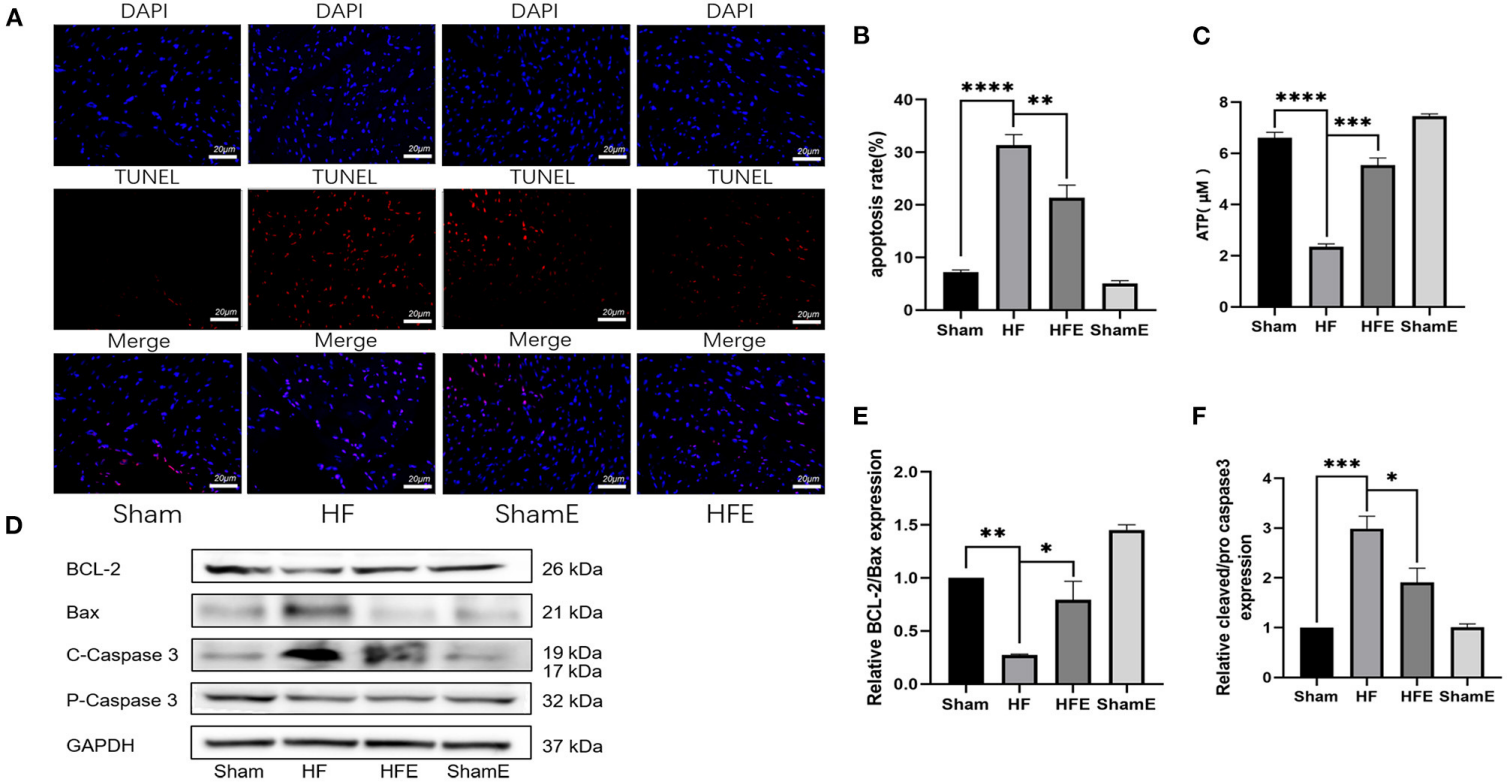
Exercise improves energy metabolism disorders and apoptosis in rats after induced myocardial infarction. **(A)** Representative TUNEL staining from rats in Sham, HF, HFE and ShamE groups, Scale = 20 μm. **(B)** ATP levels in each group; *n* = 6. ****P < 0.0001; ***P < 0.001. **(C)** Apoptosis rates in each group, ****P < 0.0001; **P < 0.01. Three random field per rat are shown. **(D)** Representative images of apoptosis-related proteins detected by WB. **(E,F)** Light density assessment of apoptosis-related proteins detected by WB. *n* = 3; ***P < 0.001, **P < 0.01, *P < 0.05.

In MI-induced HF, apoptosis is a key mediator of adverse left ventricular remodeling. In addition, alterations in ATP production are thought to play a significant role in apoptosis-induced by mitochondrial dysfunction. As shown in Figure 2, TUNEL staining revealed that the number of apoptotic nuclei was increased in the HF group, while exercise effectively decreased (*P* = 0.0099) both the number of apoptotic nuclei and the rate of apoptosis with HF (Figures 2A,B). In addition, ATP production was significantly reduced in HF compared to Sham rats, with the decrease in ATP production reversed (*P* = 0.0002) by exercise training (Figure 2C). To determine why exercise reduced the rate of apoptosis, we examined the expression of apoptosis-related proteins. As shown in Figures 2D–F, WB analysis showed that exercise could regulate the expression of apoptosis-related proteins. Specifically, compared with the Sham group, levels of cleaved/pro- caspase3 were up-regulated, while that of BCL-2/Bax was down-regulated in HF rats, with the pathological changes in apoptosis-related expression attenuated (*P* = 0.0177; *P* = 0.0146) with exercise training in the HFE group (Figures 2D–F). Therefore, it is clear that exercise may exert beneficial effects in HF by improving energy metabolism and reducing cellular apoptosis.

### Exercise-Generated BAIBA Improves Cardiac Function and Energy Metabolism Disturbance in Rats With Heart Failure

**Figure 3:**
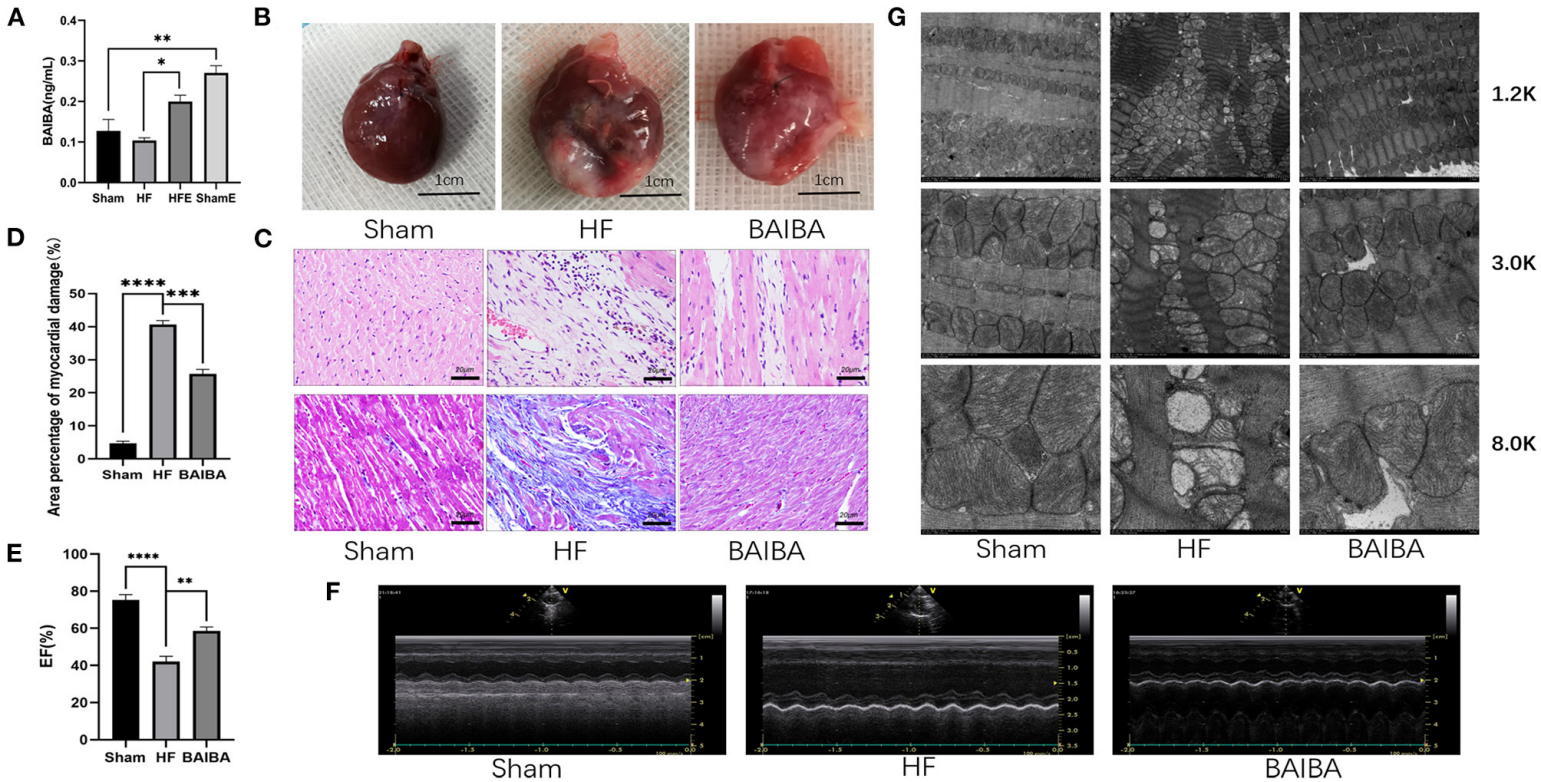
Exercise-generated BAIBA improves morphology and functioning in rats with heart failure. **(A)** BAIBA concentrations in the serum of rats from the Sham, HF, HFE, and ShamE groups; *n* = 3; **P < 0.01, *P < 0.05. **(B)** The overall appearance of the rat hearts, T scale = 1 cm. **(C)** Cardiomyocytes stained by H&E. Scale bar = 20 μm, Masson tricolor-stained cardiac fibrosis, scale = 20 μm. **(D)** Quantitative analysis of collagen deposition in the left ventricle; *n* = 3; ****P < 0.0001 ***P < 0.001. **(E)** Statistical analysis of left ventricular EF determined by echocardiography. *n* = 6; ****P < 0.0001; **P < 0.01. **(F)** M-mode echocardiography of rats in each group. **(G)** Electron micrograph (TEM) of mitochondrial morphology in a longitudinal section of ventricular muscle. Magnification, × 12,000; × 3,000, and × 80,000.

**Figure 4:**
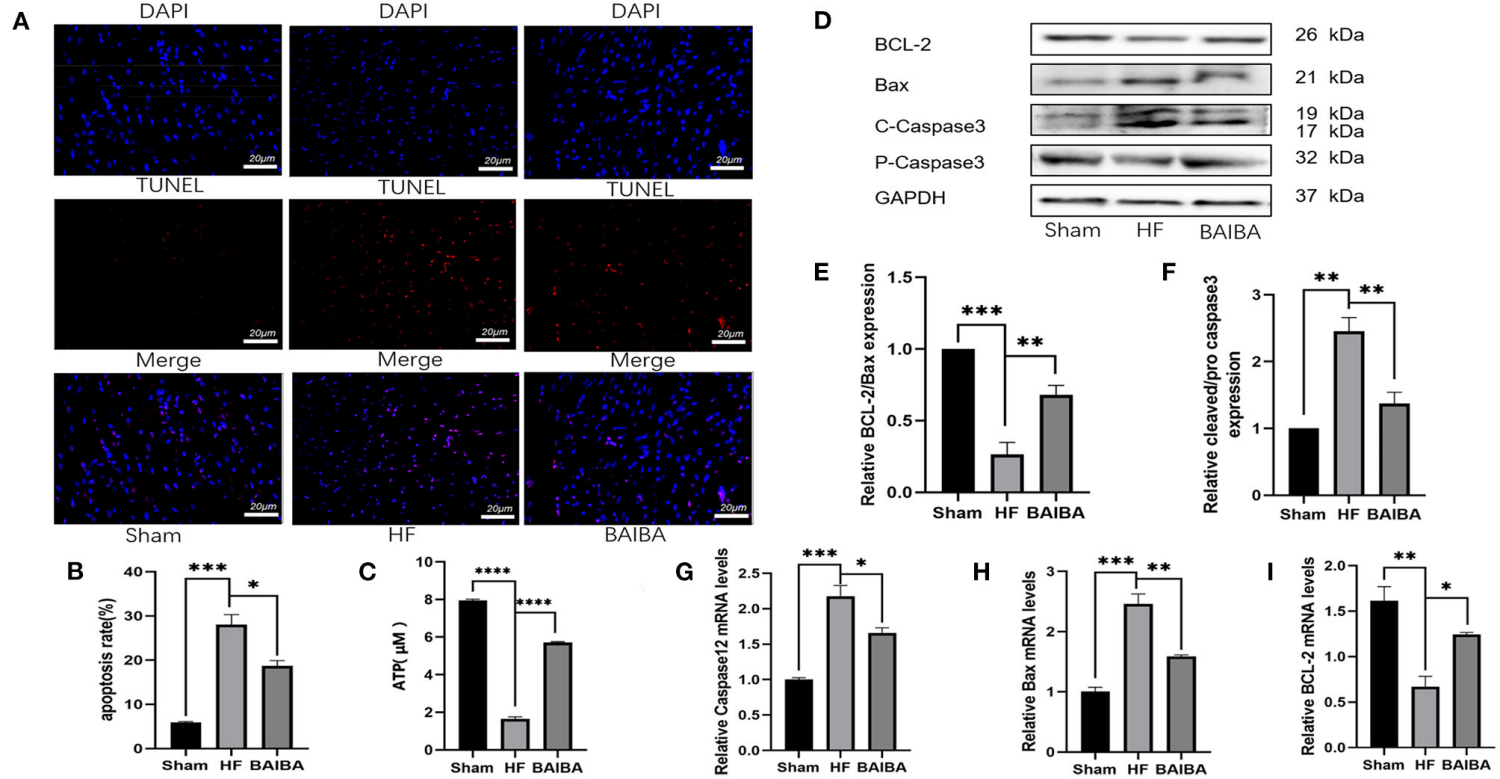
BAIBA improves energy metabolism disorders and apoptosis in rats after induced heart failure. **(A)** Representative TUNEL staining from rats in the Sham, HF and BAIBA groups, scale = 20 μm. **(B)** Apoptosis rates in each group, ***P < 0.001; *P < 0.05. Three random fields per rat are shown. **(C)** ATP levels in each group; *n* = 6. ****P < 0.0001. **(D)** Representative images of apoptosis-related proteins by WB. **(E,F)** Light density assessment of apoptosis-related proteins detected by WB. *n* = 3; ***P < 0.001, **P < 0.01. **(G–I)** Real-time quantitative PCR analysis of apoptosis-related genes. *n* = 3; ***P < 0.001, **P < 0.01, *P < 0.05.

β-aminoisobutyric acid (BAIBA) is a small molecule produced by skeletal muscle during exercise that reaches distant tissues via the bloodstream to exert beneficial physiological functions. As BAIBA appears to mediate is beneficial effects by improving energy metabolism (23, 25, 31). We sought to determine whether it may contribute to the improvements in cardiac structure and function with exercise in HF. As shown in Figure 3A, 8 weeks of exercise training increased (*P* = **0.0263**) the concentration of BAIBA in the peripheral circulation of both Sham and HF rats (Figure 3A). Given these findings, we sought to determine whether we could mimic the beneficial effects of exercise training in HF through BAIBA administration. Thus, we gave BAIBA (75 mg/kg/day) to rats by oral gavage and evaluated the cardiac function 8 weeks later to verify whether oral BAIBA could improve the cardiac structure and function of rats with HF after MI. As shown in Figure 3B, we found that 8 weeks of orally administered BAIBA improved the left ventricular morphological abnormalities in rats with HF in comparison with the HF-only group. Specifically, BAIBA was able to alleviate cardiomyocyte lysis and necrosis as well as to reduce the numbers of disordered structures (Figure 3C), reduce (*P* = 0.0001) collagen deposition (Figure 3D), and increase the EF (*P* = 0.001), FS (*P* = 0.0028) and IVDs (*P* = 0.0002) while decrease the LVIDd (*P* = 0.027) and LVIDs (*P* = 0.0001) (Figures 3E,F, Supplementary Files 3E–H) in rats with HF. TEM showed that oral administration of BAIBA could effectively reverse the morphological destruction of mitochondria caused by HF (Figure 3G). Moreover, in assessing cardiomyocyte apoptosis, TUNEL fluorescence staining showed that compared with the HF group, the numbers of apoptotic nuclei in the BAIBA-treated group were decreased (*P* = 0.011) (Figures 4A,B). Consistent with reduced apoptosis, BAIBA reversed (*P* < 0.0001) the decreases in ATP production observed with HF (Figure 4C). Importantly, WB showed that BAIBA regulated the expression of apoptosis-related proteins (Figures 4D–F) and mRNA levels (Figures 4G–I) in a similar manner to exercise, indicating that BAIBA reversed the adverse changes in apoptosis-related gene expression caused by HF.

### BAIBA Up-Regulated miR-208b to Improve Disordered Energy Metabolism and Apoptosis in Rats With HF

**Figure 5:**
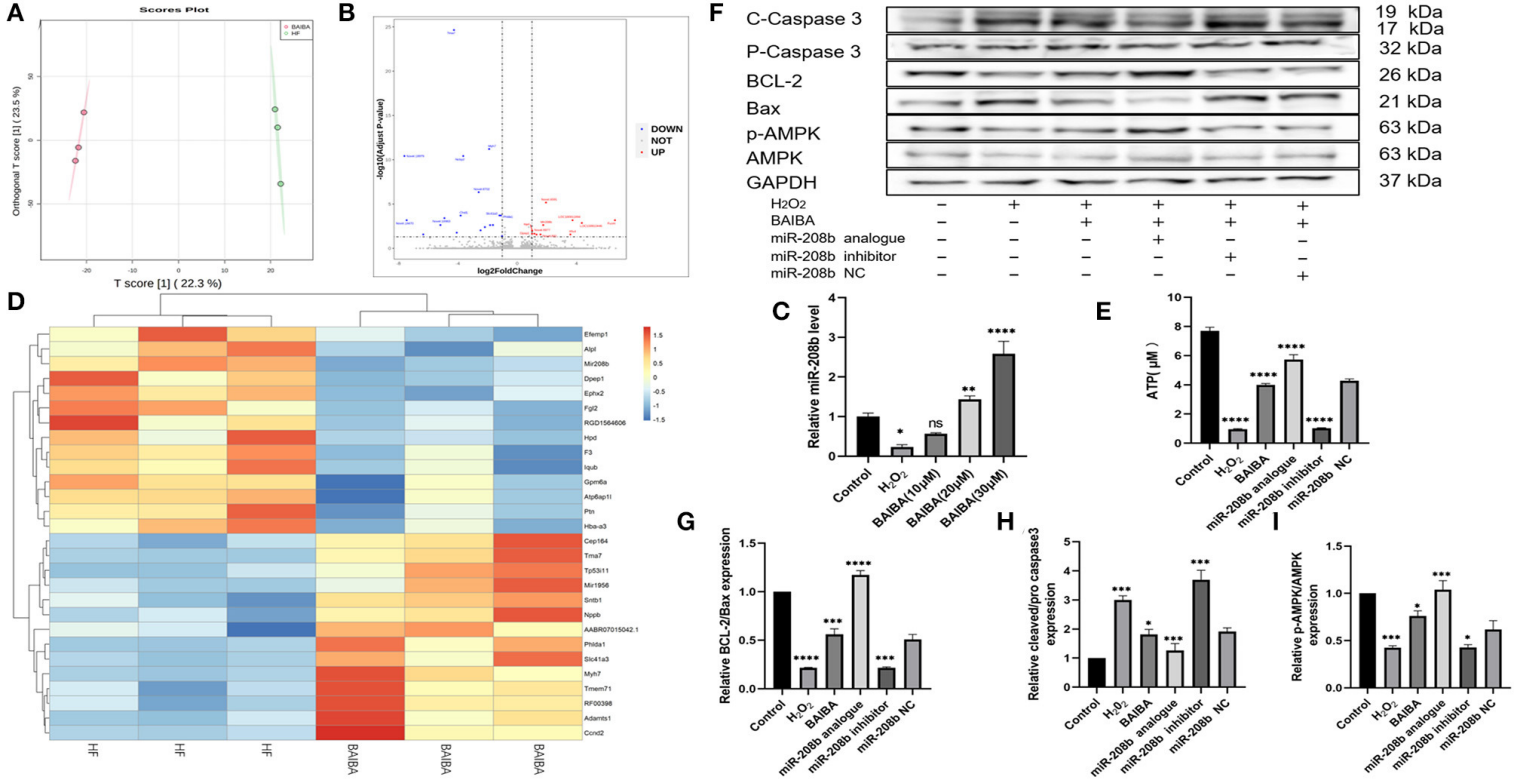
High-throughput sequencing shows that BAIBA plays an anti-apoptotic role through miR-208b. **(A)** OPLS-DA of BAIBA vs. HF group, *n* = 3. **(B)** Volcano plot of differential mRNAs. **(C)** Clustering heatmap of differential mRNA between the BAIBA and Sham groups. **(D)** Representative images of apoptosis-related proteins detected by WB. **(E)** Expression of miR-208b after stimulation with different concentrations of BAIBA vs. H_2_*O*_2_ group. *n* = 3, ****P < 0.0001; **P < 0.01; *P < 0.05. **(F)** ATP levels in each group, H_2_*O*_2_ vs. Control group; BAIBA, miR-208 analog vs. H_2_*O*_2_ group; miR-208 inhibitor vs. BAIBA group. *n* = 3. ****P < 0.0001. **(G,H)** Light density assessment of apoptosis-related proteins detected by WB. H_2_*O*_2_ vs. Control group; BAIBA, miR-208 analog vs. H_2_*O*_2_ group; miR-208 inhibitor vs. BAIBA group. *n* = 3; ****P < 0.0001, ***P < 0.001, *P < 0.05. **(I)** Light density assessment of p-AMPK detected by WB. H_2_*O*_2_ vs. Control group; BAIBA, miR-208 analog vs. H_2_*O*_2_ group; miR-208 inhibitor vs. BAIBA group. *n* = 3; ***P < 0.001, *P < 0.05.

To further understand the mechanism by which BAIBA improves energetics and exerts an anti-apoptotic effect, we next performed transcriptomic sequencing on tissue from the area surrounding the infarct in the anterior wall of the left ventricle in the HF and BAIBA groups. An orthogonal partial least squares discriminant analysis model (OPLS-DA) was used to detect the polymerization and the stability of the method. As shown in Figure 5A, the results showed a clear separation in the two groups in terms of gene expression. Specifically, RNA-sequencing revealed that 11 genes were upregulated, and 18 genes were downregulated in the BAIBA group compared to the Sham group (FC > 2.0, *P* < 0.05). Importantly, the volcano plot of differentially expressed mRNAs showed that BAIBA up-regulated the expression of miR-208b (Figure 5B), which we have previously shown to have a protective effect on myocardial apoptosis (26). Indeed, consistent with sequencing results, PCR showed alterations in miR-208b levels in response to exercise, regardless of HF or not (Supplementary File 2A). The clustering heatmap of DEGs showed differences in myocardial expression of miR-208b between the BAIBA and Sham groups (Figure 5D). Importantly, the expression of miR-208b was upregulated by BAIBA in a dose-dependent manner, with higher concentrations of BAIBA (10–30 μM) driving greater relative expression of miR-208b (Figure 5C). Moreover, BAIBA administration was found to reverse (*P* < 0.0001) the decrease in ATP concentrations in H9C2 cells caused by H_2_O_2_ (Figure 5E). Interestingly, administration of the miR-208b analog augmented BAIBA-induced cardioprotective effects, which was prevented by treatment with the miR-208b inhibitor (Figure 5F). In addition, the alterations in the expression of apoptosis-related proteins caused by H_2_O_2_ were reversed by treatment with the miR-208b analog, consistent with the effects of BAIBA, while the beneficial directional changers were attenuated by the miR-208b inhibitor (Figures 5G,H).

### BAIBA Modulates Myocardial Metabolism Through the AMPK Pathway

**Figure 6:**
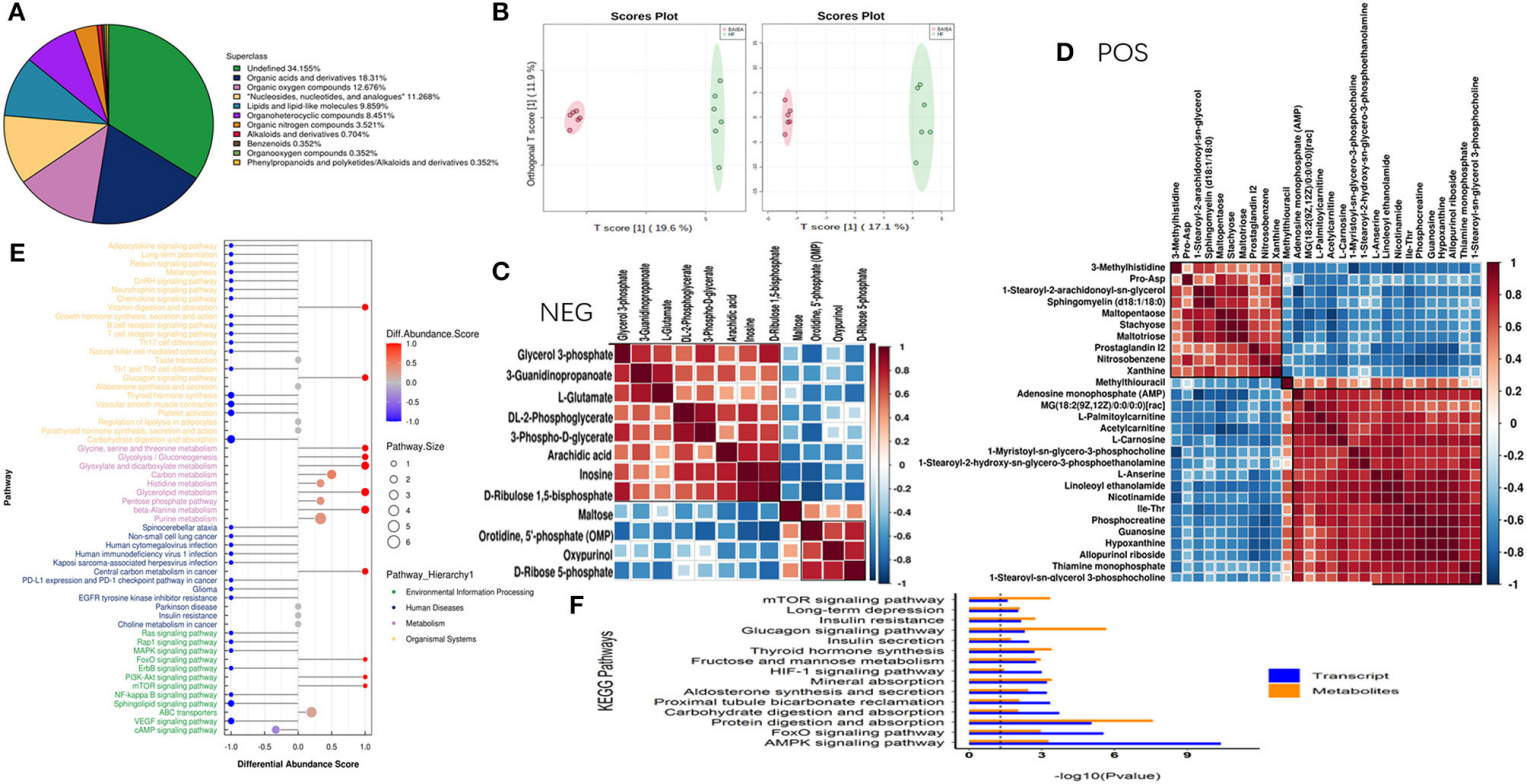
Combined transcriptomic and metabolomic analysis shows that BAIBA acts on the AMPK pathway. **(A)** The proportions of various metabolites. **(B)** OPLS-DA of BAIBA vs. HF groups, *n* = 6. **(C)** Hierarchical clustering heatmap of significantly different metabolites in the negative mode. **(D)** Hierarchical clustering heatmap of significantly different metabolites in the positive mode. **(E)** Hierarchical clustering heatmap of significantly different metabolites in the negative mode. **(F)** Significance level of the enrichment of genes and metabolites calculate by KEGG enrichment analysis.

It is clear from our study that upregulation of BAIBA and, subsequently, miR-208b improved energy metabolism and reduced apoptosis in HF rats. To further determine the specific metabolic targets of miR-208b in cardiomyocytes, a metabolomic profiling analysis was used. As shown in Figure 6A, metabolites were identified in both the positive (*n* = 183) and negative (*n* = 103) ion modes, with all metabolites classified and counted according to their chemical taxonomy and attribution. The OPLS-DA showed a clear separation between the two groups in both negative and positive ion modes (Figure 6B). Specifically, a total of 40 metabolites were found to be differentially expressed using a strict OPLS-DA Variable Importance for the Projection VIP >1 and *P*-value < 0.05 as the screening criteria (Table 1). The hierarchical clustering heatmap of the differential metabolites in the negative and positive modes is shown in Figures 6C,D. Differential abundance scores were used to analyze pathway changes in the metabolites, which showed that the differential metabolites were primarily involved in glycolysis and gluconeogenesis, glyceride metabolism, the pentose phosphate pathway, insulin resistance, purine metabolism, amino acid biosynthesis, and amino acid metabolism, histidine metabolism, arginine biosynthesis, glyoxylic acid, and dicarboxylic acid metabolism, glycine, serine, and threonine metabolism, taurine and low taurine metabolism, alanine, aspartic acid, and glutamic acid metabolism, β-glutamate, arginine and proline metabolism (Figure 6E), consistent with a role of BAIBA and miR-208b modulating myocardial metabolism. Importantly, the multi-group analysis showed that the differentially expressed genes and metabolites were mainly enriched in the AMP-activated protein kinase (AMPK) signaling pathway, which is a cellular energy sensor that plays a key role in cell growth, survival, and the maintenance of energy homeostasis. Other pathways that were enriched, to a lesser extent, in the HIF-1 signaling, FoxO signaling, fructose and mannose metabolism, insulin secretion, glucagon signaling, and insulin resistance pathways (Figure 6F).

**Table 1:** Differential metabolites between the BAIBA and Sham groups.

**Adduct**	**Name**	**VIP**	**FC**	***P*-value**	**m/z**	**rt(S)**
(M-H)-	Maltose	1.747133102	1.853496517	0.001105606	341.10984	449.316
(M-H)-	Inosine	12.68850649	0.712132158	0.001207444	267.07386	217.6715
(M+CH3COO)-	D-Ribulose 1 5-bisphosphate	1.782550319	0.408627194	0.006806923	369.00044	435.2515
(M-H)-	3-Guanidinopropanoate	2.954725215	0.706925321	0.006926241	130.06213	349.242
(M-H)-	Arachidic acid	1.684319676	0.568914743	0.007538358	311.29604	39.914
(M-H)-	DL-2-Phosphoglycerate	7.329573369	0.381760658	0.020325427	184.98581	470.9
(2M-H)-	3-Phospho-D-glycerate	1.661451592	0.236277794	0.022903901	370.98	470.953
(M-H)-	Orotidine 5′-phosphate (OMP)	1.241131943	2.081168265	0.026892841	367.01093	462.396
(M-H)-	Glycerol 3-phosphate	4.084530216	0.647349896	0.034960137	171.0064	430.0125
(M-H)-	Oxypurinol	3.882881699	2.518632379	0.035085539	151.02612	244.2205
(M+CH3COO)-	D-Ribose 5-phosphate	3.659360842	1.325621035	0.043880746	289.03359	461.66
(M-H)-	L-Glutamate	2.849537956	0.851050259	0.043943848	146.04609	396.561
(M+Na)+	Acetylcarnitine	1.462328082	0.777352931	0.000245574	226.10363	307.7345
(M+H)+	Linoleoyl ethanolamide	1.354137268	0.655316044	0.000279653	324.28769	36.2295
(M+H)+	Phosphocreatine	2.021285259	0.491283479	0.000868958	212.0419	438.9865
(M+H)+	Nicotinamide	10.74306783	0.861527316	0.000943401	123.05523	62.2415
(M+H)+	L-Carnosine	5.421254128	0.405729569	0.000965206	227.11325	422.118
(M+H)+	Hypoxanthine	11.7857247	0.749549435	0.001039384	137.04539	216.9845
(M+H)+	Guanosine	1.89912572	0.675033308	0.001314684	284.09775	262.743
(M+H)+	Ile-Thr	1.15320574	0.437272107	0.001616012	233.14837	50.792
(M+NH4)+	Stachyose	2.154557724	2.41875441	0.001949267	684.25204	488.522
(M+H-H2O)+	MG(18:2(9Z 12Z)/0:0/0:0)[rac]	3.985687426	0.713154762	0.002312178	337.2717	37.0085
(M+H-H2O)+	1-Stearoyl-2-arachidonoyl-sn-glycerol	3.795636092	1.243900004	0.003384257	627.5309	192.458
(M+NH4)+	Maltotriose	3.45554989	1.846746797	0.003497007	522.20034	449.2395
(M+CH3CN+H)+	Nitrosobenzene	1.178848398	3.325302789	0.003873997	149.07237	65.885
(2M+H)+	Allopurinol riboside	3.958330117	0.666746052	0.004621944	537.16584	216.9845
(M+H)+	1-Myristoyl-sn-glycero-3-phosphocholine	1.456332015	0.600830069	0.005324648	468.30597	200.3165
(M+H)+	Xanthine	1.574238507	1.288697953	0.005441952	153.039	219.5835
(M+Na)+	Maltopentaose	1.810989097	3.062600739	0.009029524	851.26122	507.208
(M+H)+	Prostaglandin I2	1.149081762	3.557597818	0.011067474	353.2302	161.7475
(M+H)+	Adenosine monophosphate (AMP)	1.028294798	0.585128158	0.011123783	348.06833	402.638
(M+H)+	3-Methylhistidine	2.088235504	1.488995206	0.011725758	170.09117	432.186
(M+H)+	Thiamine monophosphate	1.534209559	0.789752069	0.014247642	345.07645	490.066
(M+H)+	L-Palmitoylcarnitine	7.742755165	0.572001197	0.014438134	400.34053	175.461
(M+H)+	Sphingomyelin (d18:1/18:0)	2.537052905	1.128875942	0.016585356	731.60242	180.9475
(M+H)+	1-Stearoyl-2-hydroxy-sn-glycero-3-phosphoethanolamine	5.020219423	0.643566288	0.028989733	482.32254	198.3845
(M+H)+	Pro-Asp	1.148515883	1.075266343	0.030657874	231.09604	425.271
(M-2H+3Na)+	1-Stearoyl-sn-glycerol 3-phosphocholine	1.914627352	0.53137472	0.03432785	590.31848	184.124
(M+H-H2O)+	Methylthiouracil	1.291816999	0.788053158	0.036373882	125.01941	354.4835
(M+H)+	L-Anserine	6.031617223	0.28555341	0.04015567	241.12884	416.913

### BAIBA Increases AMPK Phosphorylation, Increases Free Fatty Acid Uptake and Oxidation Efficiency, and Improves Mitochondrial Function

**Figure 7:**
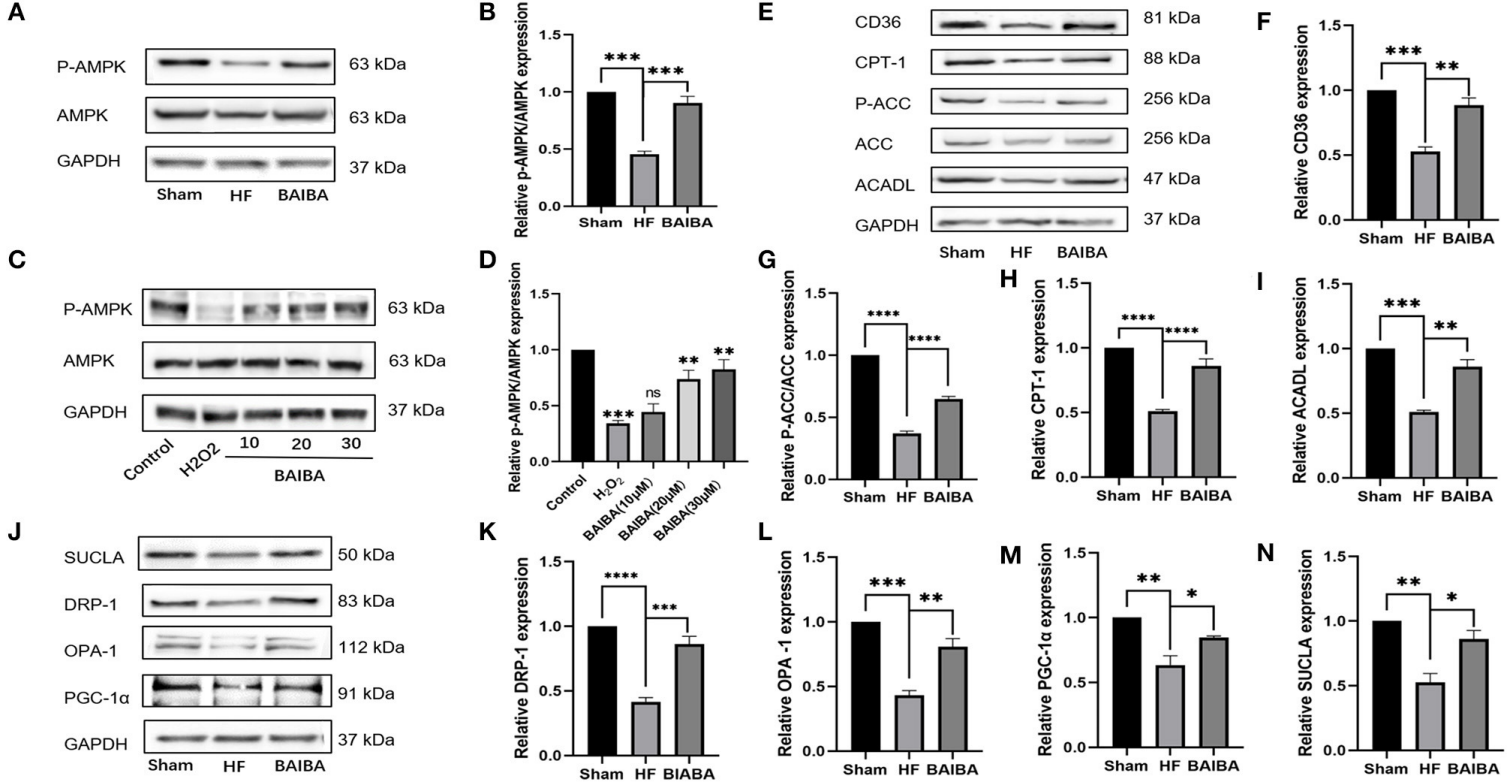
BAIBA increases cardiomyocytes AMPK phosphorylation, fatty acid intake and oxidation, and improves mitochondrial function of Heart Failure Rats. **(A)** Representative images of p-AMPK expression in the Sham, HF, and BAIBA groups detected by WB. **(B)** Light density assessment of p-AMPK expression in the Sham, HF, and BAIBA groups. *n* = 3; ***P < 0.001. **(C)** Representative images of p-AMPK expression in the Control, H_2_0_2_, BAIBA (10 mM), BAIBA (20 mM), and BAIBA (30 mM) groups detected by WB. **(D)** Light density assessment of p-AMPK expression in the Control, H_2_0_2_, BAIBA (10 mM), BAIBA (20 mM), and BAIBA (30 mM) groups. *n* = 3; ***P < 0.001, **P < 0.01. **(E)** Representative images of fatty acid uptake and oxidation-related protein expression in the Sham, HF, and BAIBA groups detected by WB. **(F–I)** Light density assessment of fatty acid uptake and oxidation-related protein expression in the Sham, HF, and BAIBA groups. *n* = 3; ****P < 0.0001; ***P < 0.001; **P < 0.01. **(J)** Representative images of mitochondrial protein expression in the Sham, HF, and BAIBA groups detected by WB. **(K–N)** Light density assessment of mitochondrial protein expression in the Sham, HF, and BAIBA groups. *n* = 3; ****P < 0.0001; ***P < 0.001; **P < 0.01; *P < 0.05.

Given the prominent enrichment of the differentially expressed in the AMPK pathway, we further interrogated the link between BAIBA administration and downstream changes in the AMPK signaling pathway. In rats with HF, oral administration of BAIBA elevated (*P* = 0.0003) the proportion of phospho-AMPK (p-AMPK) (Figures 7A,B), while in H9C2 cardiomyocytes, BAIBA also increased p-AMPK expression in the expression in a dose-dependent manner (Figures 7C,D). Transfection of the miR-208b analog amplified (*P* = 0.0001) this effect on AMPK phosphorylation, while the miR-208b inhibitor counteracted (*P* = 0.0222) the effect (Figures 5F,I). Taken together, these findings suggest that the phosphorylation of AMPK by BAIBA may be mediated by miR-208b. As a main metabolic switch, activated AMPK can increase the flexibility of metabolic substrates and improve mitochondrial function through mitochondrial biogenesis and quality control. Thus, we next performed WB analysis of downstream AMPK proteins in HF rat models. Fatty acids are an important metabolic substrate in the heart, and we therefore assessed changes in a number of these key enzymes in fatty acid oxidation (FAO), including carnitine palmitoyltransferase-1 (CPT-1), acyl-CoA dehydrogenase long chain (ACADL), and acetyl-CoA carboxylase (ACC), using WB (Figure 7E). In quantifying changes in these enzymes, compared with the HF group, BAIBA increased (*P* = 0.0014) the expression of the fatty acid uptake-related protein CD36 (Figure 7F). Moreover, BAIBA increased the expression of p-ACC (*P* < 0.0001) (Figure 7G), CPT-1 (*P* < 0.0001) (Figure 7H), and ACADL (*P* = 0.0015) (Figure 7I), all of which would increase fatty acid uptake and oxidation efficiency. At the same time, BAIBA also increased the biogenesis of mitochondria and improved their functioning (Figure 7J). Mitochondrial fusion and fission form the core of mitochondrial quality control. Our results showed that BAIBA ameliorated (*P* = 0.002; *P* = 0.0004) the reductions in optic nerve atrophy 1 (OPA1) and dynamic protein-associated protein 1 (Drp1) expression, thus improving mitochondrial quality (Figures 7K,L). Moreover, BAIBA was found to increase (*P* = 0.0288) the expression of peroxisome proliferator-activated receptor (PPAR) γ coactivator-1 α (PGC-1α), which is a strong activator of cardiac mitochondrial biogenesis (Figure 7M). SUCLA2 is a core enzyme of the tricarboxylic acid cycle (TCA), participating in acetyl-CoA oxidation; BAIBA attenuated (*P* = 0.0114) the decreased SUCLA expression in rats in the HF group (Figure 7N). Therefore, our findings suggest BAIBA mediates its beneficial effects on cardiac energetics by increasing AMPK phosphorylation, fatty acid intake and oxidation, and improving mitochondrial function.

### The Protective Effects of BAIBA on Apoptosis, Disordered Energy Metabolism, Mitochondrial Function, and FA Uptake and Oxidation Are Offset by AMPK Inhibition

**Figure 8:**
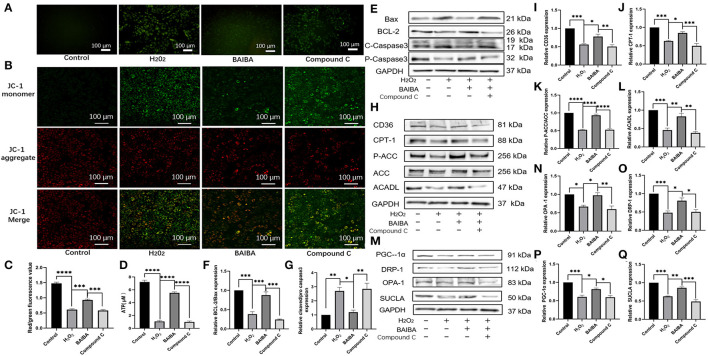
Effects of BAIBA administration on apoptosis, energy metabolism disorders, mitochondrial function, and fatty acid uptake and utilization. **(A)** ROS production in Control, H_2_*O*_2_, BAIBA, and Compound C-treated cells, the scale bar in each image is 100 μm. **(B)** Representative ΔΨ m staining determined by measuring changes in JC-1-derived fluorescence from red (high potential, J-aggregates) to green (low potential, monomeric) using confocal microscopy; data are representative of three independent experiments. **(C)** Statistical analysis of JC-1 red fluorescence percentage; *n* = 5 wells. ***P < 0.0001, ****P < 0.0001; **(D)** ATP levels in each group; *n* = 3. ****P < 0.0001; **(E)** Representative images of apoptosis-related proteins of each group. **(F,G)** Light density assessment of apoptosis-related protein expression. *n* = 3; ***P < 0.001, **P < 0.01, *P < 0.05. **(H)** Representative images of fatty acid uptake and oxidation-related protein expression in each group. **(I–L)** Light density assessment of fatty acid uptake and oxidation-related protein expression. *n* = 3; ****P < 0.0001; ***P < 0.001; **P < 0.01. *P < 0.05. **(M)** Representative images of mitochondria related protein expression of each group. **(N–Q)** Light density assessment of mitochondria related protein expression, *n* = 3; ***P < 0.001; **P < 0.01; *P < 0.05.

Our results suggest that the protective effect of BAIBA was mediated by the AMPK pathway. To further interrogate how activation of this pathway attenuates apoptosis, we began by assessing the impact of BAIBA on ROS production, which is known initiator of cardiomyocyte apoptosis. As shown in Figure 8A, ROS production was increase in the H_2_O_2_-treated group compared with the controls, consistent with a link to myocardial apoptosis. Importantly, treatment with BAIBA reversed this effect by reducing ROS production, while treatment with the AMPK inhibitor, Compound C, counteracted the BAIBA-mediated action (Figure 8A). Moreover, mitochondrial membrane potential staining showed similar directional changes with our treatment groups. Specifically, BAIBA treatment counteracted the drop in mitochondrial membrane potential caused by H_2_O_2_, which was counteracted (*P* = 0.0002) by treatment with Compound C (Figures 8B,C). Treatment with Compound C also reversed (*P* < 0.0001) the protective effects of BAIBA on ATP production (Figure 8D). Consistent with above, the BAIBA-mediated reversal of H_2_O_2_-induced changes in apoptosis-related protein expression was also offset by the AMPK inhibitor (Figures 8E–G). Analysis of the expression of proteins associated with fatty acid uptake and β-oxidation (Figures 8H–L) as well as mitochondrial proteins (Figures 8M–Q) further confirmed these findings, demonstrating that BAIBA mediates its cardioprotective effects through AMPK and that AMPK inhibition reverses the protective effect of BAIBA on the expression of these proteins.

## Discussion

The heart is an energy-consuming organ and requires sufficient ATP to maintain its pump function. Normal cardiomyocytes have metabolic flexibility. Various metabolic substrates including fatty acids, carbohydrates (glucose and lactates), ketones, and amino acids enter the tricarboxylic acid cycle to form ATP, allowing the maintenance of normal cardiac contraction. Mitochondria play important roles in the production of ATP. By contrast, a loss of metabolic substrate flexibility caused by mitochondrial dysfunction may be a key factor in heart failure, leading to metabolic and oxidative stress as well as increased cellular apoptosis, forming a vicious cycle. Our results showed that in rats with HF after MI, the mitochondria were arranged in a disordered fashion, with swollen mitochondrial crests and many absent cristae, and there was significant mitochondrial vacuolation. As a result, the production of ATP decreased, which accelerated the progression of HF. Therefore, mitochondrial function is a crucial therapeutic target for heart failure (2, 32).

Moderate exercise is known to have a beneficial effect on heart function in patients with HF (9). Moderate continuous training is efficient, safe, and well tolerated by HF patients, and it is recommended by the Heart Failure Association Guidelines (15). Cardiac energy metabolism can be partially restored through exercise due to improvements in oxidation ability and by restoring energy transfer. Exercise can also restore cardiac autophagic flux in heart failure. This is related to improved mitochondrial quality control, bioenergetics, and cardiac function (16). Our results showed that in rats with HF after MI, the EF% of the exercise group was superior to that of the HF group. Despite being forced exercise, which may be associated with stress responses (33), 8 weeks of treadmill training alleviated the degree of cardiomyocyte apoptosis surrounding the infarcted region and improved mitochondrial morphology. The protective function of exercise in HF patients undergoing continuous aerobic exercise training is primarily determined by the total energy expenditure, including the training intensity, duration, and frequency of the training program. However, a considerable number of patients with HF are unable to undertake effective exercise due to circulatory disorders and skeletal muscle mitochondrial dysfunction, among other reasons. For these patients, their recovery can potentially be improved by the identification of the targets of exercise-induced protection and using these to find alternative treatments.

BAIBA is a small molecule (103.6 Da) produced by skeletal muscles during exercise and mediates the beneficial effect of exercise from skeletal muscle to other tissues and organs via the endocrine system (19). BAIBA activates the fatty acid β-oxidation pathway in the liver, converts white adipose tissue to brown fat (25), improves insulin resistance and skeletal myositis by the autocrine/paracrine systems, prevents diet-induced obesity (34), and protects against metabolic dysfunction in type 2 diabetes (22). Here, it was found that the production of BAIBA increased after exercise in both the control group and HF rats, which may underlie the cardioprotective effects of exercise in HF. Importantly, we found that 8-weeks of BAIBA administration in rats with HF improved cardiac function, reversed metabolic stress, and reduced apoptosis. Moreover, transcriptomic analyses revealed this protective action of BAIBA was found to be linked to increased expression of miR-208b, which is a heart-specific miRNA encoded on intron 27 of the gene encoding α-MHC that is essential for the expression of genes involved in cardiac contractility and fibrosis. An increased plasma concentration of miR-208b has been linked to myocardial injury and may serve as a biomarker of acute myocardial infarction (35). Nonetheless, previous studies have demonstrated miR-208b may serve a protective role in myocardial fibrosis after infarction through GATA4 signaling (36). Consistent with a cardioprotective role of miR-208b in the current study, we have previously shown that miR-208b has a protective effect on myocardial apoptosis (26). Specifically, our findings confirm that BAIBA increases the expression of miR-208b in a dose-dependent manner, and that the miR-208b analog is a downstream mediator of the protective effects on left ventricular remodeling and function through the reduction of metabolic stress and apoptosis caused by mitochondrial dysfunction in rats with HF. Indeed, metabolomic profiling linked the protective effects to differential regulation of metabolites involved in glycolysis, gluconeogenesis, amino acid biosynthesis, and other metabolic pathways, consistent with the maintenance of mitochondrial energetics being important being a crucial therapeutic target in HF. Importantly, we identified that the protective effect of BAIBA on the heart in HF is linked to the AMPK signaling pathway, with both BAIBA and miR-208b increasing AMPK phosphorylation in a concentration-dependent manner. As miR-208b analog transfection enhanced AMPK activation by BAIBA, and the miR-208b inhibitor counteracted this effect, our findings suggest a novel cardioprotective signaling cascade in which activation of AMPK by BAIBA may be mediated by miR-208b.

Our *in vivo* findings of a BAIBA-mediated increases in AMPK supports the notion that reducing cardiomyocyte metabolic stress is important for HF treatment and outcomes. As a cellular energy sensor and metabolic switch, AMPK plays a key role not only in cell growth and survival but also in systemic energy homeostasis (37). Moreover, AMPK mediates mitochondrial fission to cope with energy stress, which would be increased in HF. Our findings support a mechanistic framework by which BAIBA reduces cardiomyocyte metabolic stress and apoptosis caused by mitochondrial dysfunction through the miR-208b/AMPK pathway. Indeed, our results showed that BAIBA can not only increase the expression of AMPK, but can effectively improve the expression of proteins related to fatty acid uptake and oxidation, which is known to have beneficial effects in HF models (38, 39). Mechanistically, BAIBA promotes fatty acid uptake by cardiomyocytes by increasing the translocation of the fatty acid transporter CD36 to the membrane (40). In addition, the rate-limiting step of β-oxidation is the CPT1-mediated mitochondrial transmembrane transfer of fatty acids. Moreover, AMPK inhibits the production of malonyl-CoA carboxylase (ACC) by phosphorylation, increases the activity of CPT1, and increases the efficiency of myocardial fatty acid β-oxidation (41). As BAIBA increases CPT-1 levels in the context of heart failure and thereby improves the efficiency of fatty acid oxidation and attenuates metabolic stress (42). Indeed, we found that BAIBA effectively improved the expression of mitochondrial proteins. As the power houses that maintain cardiac energy supply, mitochondrial dysfunction is a prominent mediatory of pathological processes that can drive adverse cardiac remodeling, including redox imbalance, protein modification, ROS signaling, ion homeostasis, and inflammation (43, 44). However, our findings suggest BAIBA may prevent mitochondrial dysfunction by upregulating the expression of OPA1 and Drp1. Mitochondrial fusion and fission are the core of mitochondrial quality control and the fusion of the mitochondrial inner membrane is dependent on OPA1, which maintains closure of the crest junction, preventing cytochrome c release and apoptosis (45). Thus, reduced expression of OPA1 in dysfunctional mitochondria is accompanied by the destruction and loss of the mitochondrial crest and loosening of the mitochondrial junction, leading to apoptosis (46, 47). Moreover, Drp1 is a GTP enzyme that mediates mitochondrial fission (48) which promotes autophagy of damaged mitochondria (49). By contrast, reduced levels of Drp1 results in mitochondrial elongation and accumulation of damaged mitochondria, together with an inhibition of mitochondrial autophagy, leading to mitochondrial dysfunction and cardiomyocyte apoptosis (50). Thus, the improvement in mitochondrial function could increase the stability of the mitochondrial membrane potential and optimize the morphology of the mitochondrial crest, thus avoiding cytochrome c release and ROS accumulation, and ultimately preventing apoptosis. Taken together, our findings suggest that in patients with HF, the pharmacological activation of AMPK with BAIBA could be used as a therapeutic strategy to improve cardiac energy supply. While SGLT2, metformin, statins, and trimetazidine have received significant attention in the treatment of cardiovascular diseases because of their activation of AMPK (51–54), our findings suggest that BAIBA administration could be an alternative therapeutic approach to reduce metabolic stress and mitochondrial dysfunction in HF.

In addition to regulating the metabolism of the failing heart, AMPK can also mediate a variety of physiological signaling pathways (55). AMPK promotes autophagy directly through phosphorylation of the Unc-51-like kinase 1 (ULK1) kinase complex (56), and indirectly enhances autophagy through mTORC1 inactivation (57). The inhibition of mTOR affects protein synthesis, reducing the accumulation of unfolded proteins and thus reducing endoplasmic reticulum stress (58). AMPK activation can also reduce inflammation and cell death by inhibiting JNK and NF-κB, thereby protecting cardiomyocytes (59).

Our study is not without limitations. While our findings show the cardioprotective effect of BAIBA is mediated through an miR-208b/AMPK-mediated pathway, we cannot rule out that other pathways may contribute to the protective effect on cardiac structure and function. Moreover, we did not use double staining to indicate which cell type in heart tissue is subjected to apoptosis. Co-staining cardiomyocytes with TUNEL staining would be more convincing for the presentation of the results, though it is well-established that MI models of HF induce cardiomyocyte apoptosis. In addition, we used GAPDH as an internal control for normalizing expression. However, apoptosis caused by mitochondrial dysfunction may be accompanied by changes in glycolysis. To overcome this limitation, we further monitored the relative levels of β-actin in our samples, with our results indicating the relative level of GAPDH is almost equal to that of β-actin (Supplementary File 2).

In conclusion, this study confirmed that exercise-generated BAIBA increased AMPK phosphorylation through the expression of miR-208b to reshape the energy metabolism of rats with HF, improving mitochondrial dysfunction and reducing apoptosis. But for patients with heart failure, whether BAIBA can be used as an alternative for exercise therapy still requires more studies.

## Conclusion

Exercise-generated BAIBA can reduce cardiomyocyte metabolic stress and apoptosis induced by mitochondrial dysfunction through the miR-208b/AMPK pathway.

## Data Availability Statement

The data presented in the study are deposited in the MetaboLights repository, accession number MTBLS3927; And GEO repository, accession number GSE193432.

## Ethics Statement

Animal experiments were approved by the Ethics Committee of the First Hospital of Jilin University. The ethics number is 20210710. Written informed consent was obtained from the patient for the publication of this case report and any accompanying images.

## Author Contributions

PY is the chief investigator, conceived the study, led the proposal, and protocol development. WWC, MY, and JSL contributed to study design. YNY performed experiments, analyzed and interpreted the data, and drafted the manuscript. HS contributed resources, designed research, in supervised the project, and reviewed the manuscript. All authors read and approved the final manuscript.

## Funding

This work was supported by the National Natural Science Foundation of China, No. 81570360.

## Conflict of Interest

The authors declare that the research was conducted in the absence of any commercial or financial relationships that could be construed as a potential conflict of interest.

## Publisher's Note

All claims expressed in this article are solely those of the authors and do not necessarily represent those of their affiliated organizations, or those of the publisher, the editors and the reviewers. Any product that may be evaluated in this article, or claim that may be made by its manufacturer, is not guaranteed or endorsed by the publisher.
