# Supplementary material for: Exercise-Generated β-Aminoisobutyric Acid (BAIBA) Reduces Cardiomyocyte Metabolic Stress and Apoptosis Caused by Mitochondrial Dysfunction Through the miR-208b/AMPK Pathway
Source: Front Cardiovasc Med. 2022 Feb 25;9:803510. doi: 10.3389/fcvm.2022.803510 (PMC8915946; doi:10.3389/fcvm.2022.803510)
Supplement: Supplementary file 1 [file Data_Sheet_1.docx]

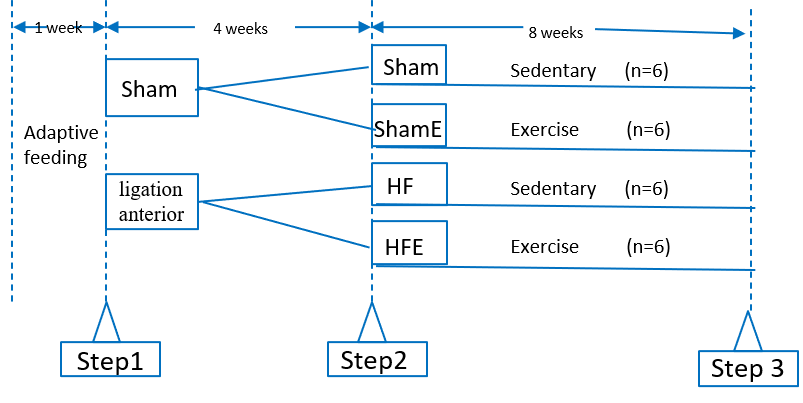


**Supplementary document 1：**

**Step1:** Ligation the anterior descending coronary artery to create the rat HF model. The Sham group was threaded only without ligation.

**Step2:** All surviving rats were examined by echocardiography and were divided into groups according to cardiac function. HFE group and ShamE group rats were treated with treadmill exercise, while the Sham and HF groups were sedentary.

**Exercise training program:** Rats in HFE and ShamE group were involving 10-20 min acclimation, 0.3-0.6 km/h, and 0% incline. After three days’ acclimation, the maximum exercise intensity was tested, starting at 0.3 km/h and increasing by 0.3 km/h every 3 min until exhaustion. The rats were run on the treadmill for eight weeks (50% to 60% of maximum intensity) ,60 min/day, 5 days/week, and at a 0% incline.

**Step3:** All surviving rats were again examined by echocardiography, after which the hearts were removed and frozen in liquid nitrogen until used for transcriptomic and metabolomic sequencing and and biochemical analysis.


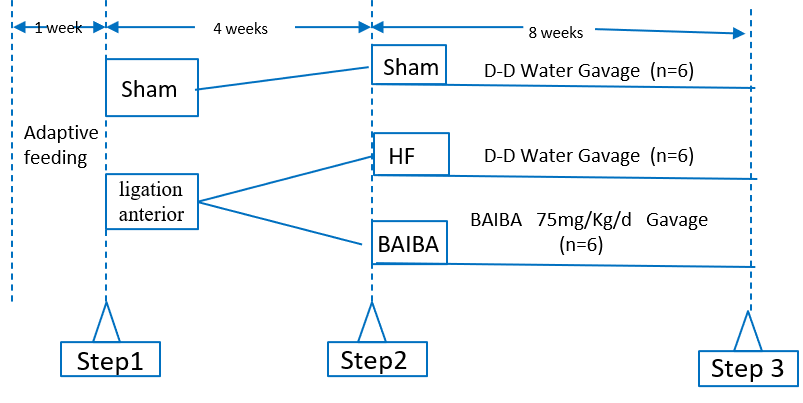


**Step1:** Ligation of the anterior descending coronary artery to create the rat HF model. The Sham group was threaded only without ligation.

**Step2:** All surviving rats were examined by echocardiography and divided into groups according to cardiac function. The Sham and HF groups were treated with BAIBA（75mg/Kg/day）.

**Step3:** All surviving rats were again examined by echocardiography, after which the hearts were removed and stored in liquid nitrogen for biochemical analysis.
