# Supplementary material for: Exercise-Generated β-Aminoisobutyric Acid (BAIBA) Reduces Cardiomyocyte Metabolic Stress and Apoptosis Caused by Mitochondrial Dysfunction Through the miR-208b/AMPK Pathway
Source: Front Cardiovasc Med. 2022 Feb 25;9:803510. doi: 10.3389/fcvm.2022.803510 (PMC8915946; doi:10.3389/fcvm.2022.803510)
Supplement: Supplementary file 2 [file Data_Sheet_2.docx]

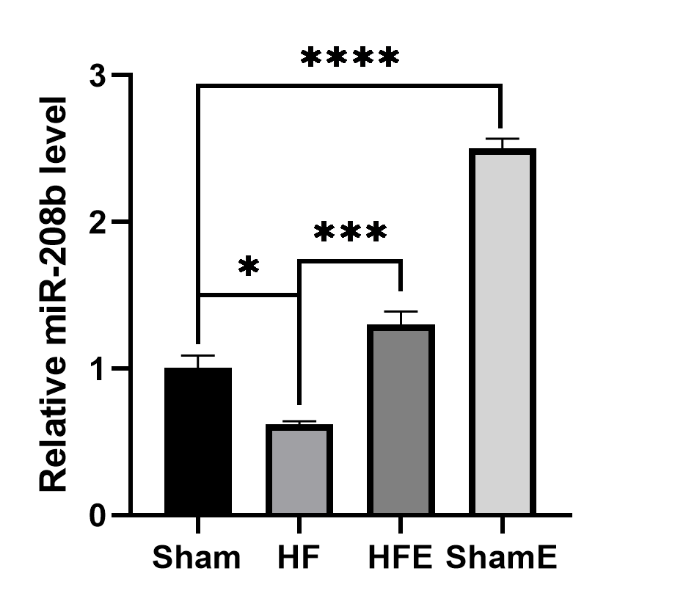


**Supplementary file 2-A:** Expression of miR-208b in Sham, HF, HFE and ShamE groups, n=3, ****P＜0.0001；***P＜0.001; *P＜0.05.


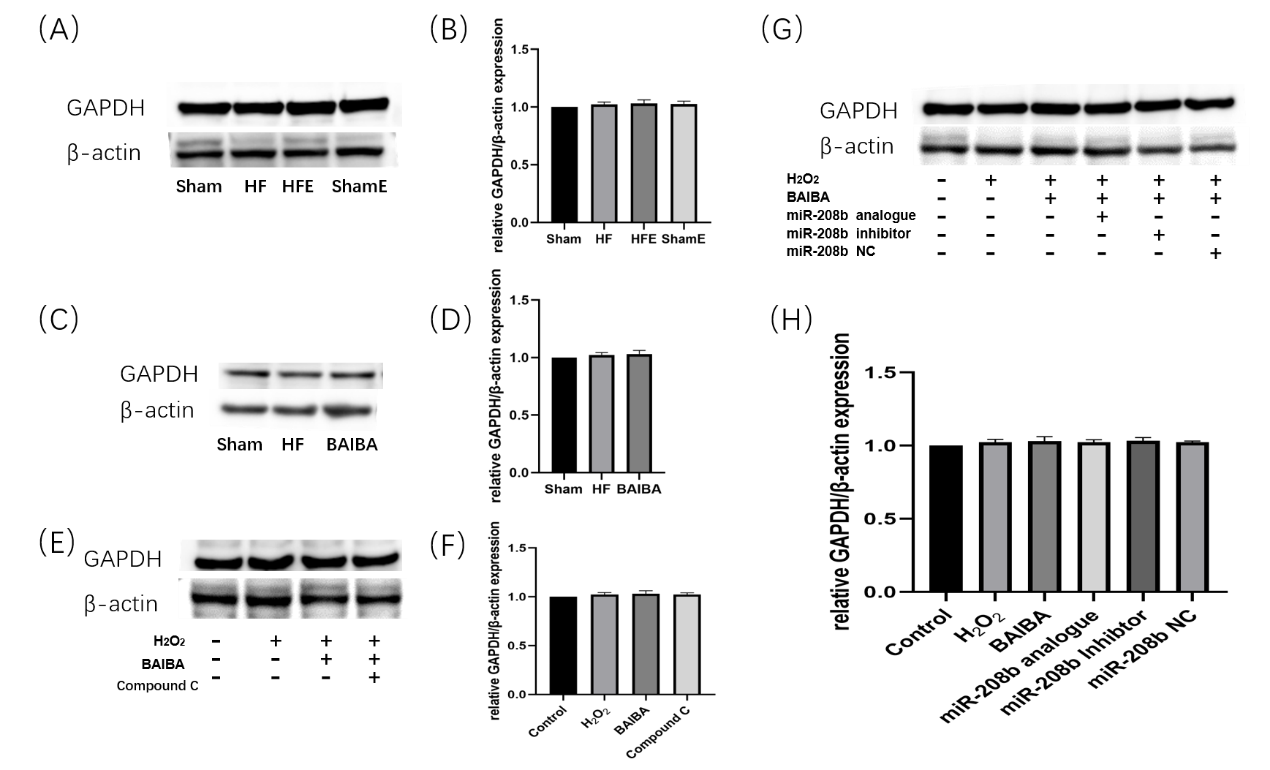


**Supplementary file 2-B:** (A) Representative images of GAPDH and β-Actin proteins by WB in Sham,HF,HFE and ShamE group, (B) Light density assessment of control proteins detected by WB. n=3; P＞0.05.(C) Representative images of control proteins by WB in Sham, HF and BAIBA group, (D) Light density assessment of control proteins detected by WB. n=3; P＞0.05. (E) Representative images of control proteins by WB in Control, H_2_0_2_, BAIBA, and compound C-treated cells; (F) Light density assessment of control proteins detected by WB. n=3; P＞0.05. (G) Representative images of control proteins by WB in Control, H_2_0_2_, BAIBA, miR-208b analogue; miR-208b inhibitor and miR-208b NC group cells; (F) Light density assessment of control proteins detected by WB. n=3,P＞0.05.
