# Supplementary material for: Exercise-Generated β-Aminoisobutyric Acid (BAIBA) Reduces Cardiomyocyte Metabolic Stress and Apoptosis Caused by Mitochondrial Dysfunction Through the miR-208b/AMPK Pathway
Source: Front Cardiovasc Med. 2022 Feb 25;9:803510. doi: 10.3389/fcvm.2022.803510 (PMC8915946; doi:10.3389/fcvm.2022.803510)
Supplement: Supplementary file 3 [file Data_Sheet_3.docx]

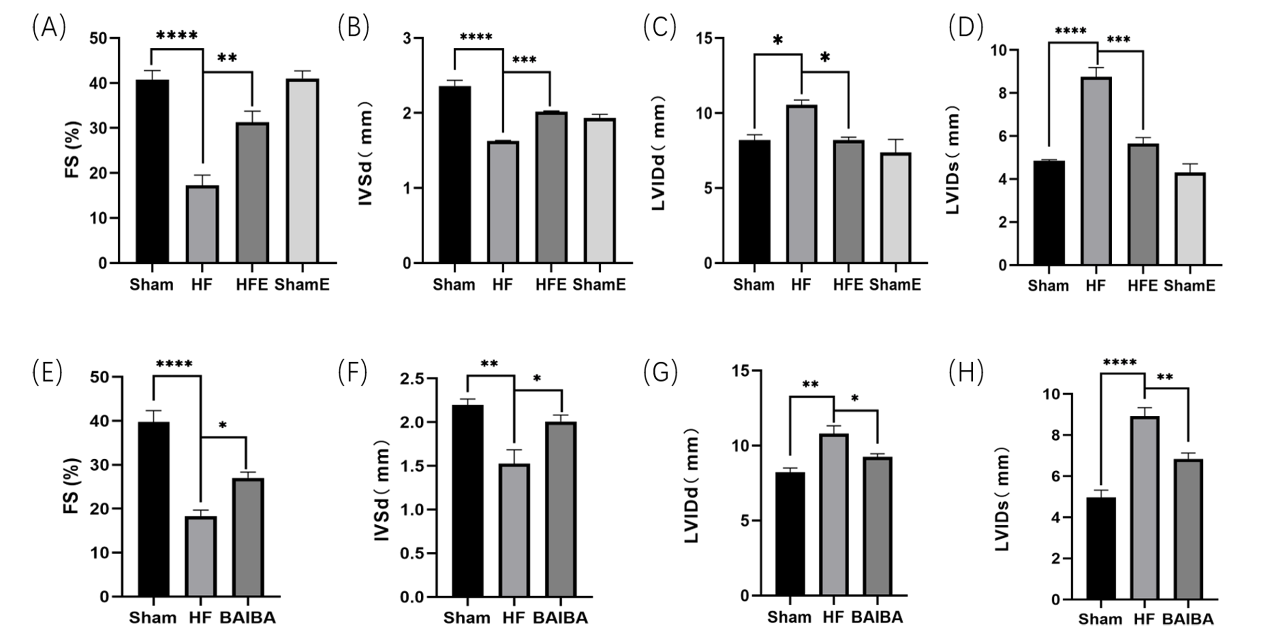


**Supplementary file 3：Exercise and BAIBA improved the cardiac function of rats with heart failure after myocardial infarction. (A)** Statistical analysis of fractional shortening (FS%) determined by echocardiography in Sham, HF, HFE and ShamE groups. n=4; **** P＜0.0001；**P＜0.01. **(B)** Statistical analysis of interventricular septal thickness (IVDs) determined by echocardiography in Sham, HF, HFE and ShamE groups. n=4; **** P＜0.0001；***P＜0.001. **(C)** Statistical analysis of left ventricular end diastolic diameter (LVIDd) determined by echocardiography in Sham, HF, HFE and ShamE groups. n=4; *P＜0.05. **(D)** Statistical analysis of Left ventricular end systolic diameter (LVIDs) determined by echocardiography in Sham, HF, HFE and ShamE groups. n=4; **** P＜0.0001；***P＜0.001. **(E)** Statistical analysis of fractional shortening (FS%) determined by echocardiography in Sham, HF, and BAIBA groups. n=4; **** P＜0.0001；*P＜0.05. **(F)** Statistical analysis of interventricular septal thickness (IVDs) determined by echocardiography in Sham, HF and BAIBA groups. n=4; ** P＜0.01；*P＜0.05. **(G)** Statistical analysis of left ventricular end diastolic diameter (LVIDd) determined by echocardiography in Sham, HF and BAIBA groups. n=4; ** P＜0.01；*P＜0.05. **(H)** Statistical analysis of Left ventricular end systolic diameter (LVIDs) determined by echocardiography in Sham, HF and BAIBA groups. n=4; **** P＜0.0001；**P＜0.01.
